# Nature of low-frequency noise in homogeneous semiconductors

**DOI:** 10.1038/srep18305

**Published:** 2015-12-17

**Authors:** Vilius Palenskis, Kęstutis Maknys

**Affiliations:** 1 Vilnius University, Faculty of Physics, Saulėtekio al. 9, LT-10222 Vilnius, Lithuania

## Abstract

This report deals with a 1/*f* noise in homogeneous classical semiconductor samples on the base of silicon. We perform detail calculations of resistance fluctuations of the silicon sample due to both a) the charge carrier number changes due to their capture–emission processes, and b) due to screening effect of those negative charged centers, and show that proportionality of noise level to square mobility appears as a presentation parameter, but not due to mobility fluctuations. The obtained calculation results explain well the observed experimental results of 1/*f* noise in Si, Ge, GaAs and exclude the mobility fluctuations as the nature of 1/*f* noise in these materials and their devices. It is also shown how from the experimental 1/*f* noise results to find the effective number of defects responsible for this noise in the measured frequency range.

The 1/*f* noise problem in various electronic devices has been investigated over 80 years, and over 60 years in solids, but the origin of the 1/*f* noise is still open on discussions. It was directly shown that 1/*f* noise in homogeneous materials is due to its resistance *R* fluctuations Δ*R*(*t*) at equilibrium conditions^1^,^2^. The special measurements prove that 1/*f* noise is not generated by the current. In conventional measurements the current is only necessary to transform the already existing resistivity (conductivity) fluctuations into voltage fluctuations that can be measured. In^2^, it is also shown that 1/*f* noise in nonlinear elements (diodes or transistors) is due to fluctuation of the conversion transconductance. A problem of spectral density of the resistance fluctuations dependence on frequency has been widely discussed for different materials in many works^3^,^4^,^5^,^6^,^7^,^8^,^9^,^10^,^11^,^12^,^13^,^14^,^15^,^16^,^17^ and others. Usually it has been considered that for classical semiconductors and their devices 1/*f* noise is a result of superposition of Lorentzian type spectra due to different generation-recombination or capture-emission of charge carrier processes with very wide distribution of relaxation times^8^,^9^.

F. N. Hooge systematized the 1/*f* noise measurement results of spectral density of resistance fluctuations *S*_*R*_ for linear resistances *R* in such relation 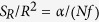 (here *N* is the total free carrier number in the sample, *f* is the measurement frequency)^3^,^18^. The *α* values were scattered, but it was accepted as an average value 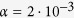. There was no reason to assume that parameter *α* is a constant. Later appears that *α* depends on the quality of the sample material. In high quality material *α* can be more than 3 orders of magnitude lower than the originally proposed value^19^. Many experiments show that the noise is only proportional to 1/*N*, when *N* is changed by changing the volume of the sample. Damage and various defects of the sample material have a strong influence to the *α* value, and it may increase *α* value by many orders of magnitude^14^.

The dimensions length, width, and thickness do not change α value, providing that 1/*f* noise is a bulk effect. Thus, the theories on the ground of the surface effect have been refused^18^, but there is a positive evidence that surface 1/*f* noise exists too: experimental data in MOSTs are better explained by surface effects than by bulk effects^20^,^21^.

The same relation 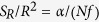 can be applicable to metals: it is clear that for noise studies in metals very small samples are required. Experiments on point contacts^22^,^23^ and thin films^24^ show that this relation does indeed hold, and *α* has the same order of magnitude as in semiconductors.

On the ground of experimental results that α value in homogeneous materials such Si, Ge, GaAs and others is proportional to square mobility *μ*^2^, it has been stated that 1/*f* noise is caused by mobility fluctuations of the free charge carriers due to lattice scattering^4^,^25^. It has been considered that scattering cross-section fluctuates slowly with a 1/*f* spectrum, and this idea is live till now^26^. It is very strange because the scattering processes are very fast, for example, the relaxation times for different scattering mechanisms in silicon are in the range from 10^−14^ s to 10^−12^ s^27^,^28^.

In this work we present calculations results for resistance fluctuations on the base of the silicon sample due to charge carrier number changes caused by their capture–emission process, and will show that flicker noise level proportionality to the square mobility does not mean that the 1/*f* noise origin is mobility fluctuations due to charge carrier lattice scattering.

## Results

### Calculation of the resistance fluctuations

**Figure 1:**
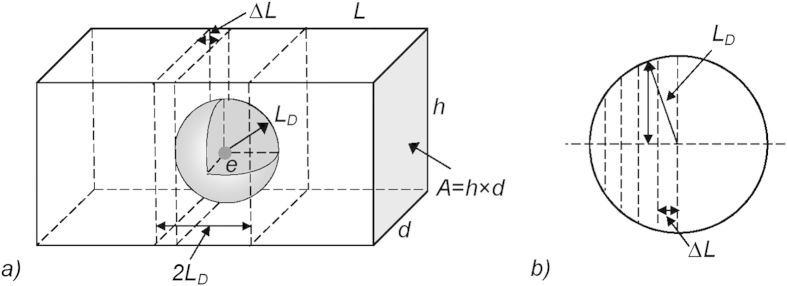
Schematic pattern for evaluation of the resistance fluctuation of the investigated sample (a) due to forming of the screened volume (Debye sphere (b)). The value of the step Δ*L* has been chosen in such a way that volume of the bar within the length 2*L*_D_ was equal to the sum of the volume of the Debye sphere and the volume of the rest part in the range 2*L*_D_ without this sphere.

For simplicity we study a silicon sample of the volume 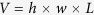 (here *h*, *w* and *L* is the height, the width and the length of the sample, respectively), inside of it there are *N* free electrons and a single deep neutral capture center (Fig. 1). The resistance of this sample is


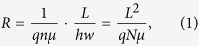


where *q* is the electric charge of the electron, 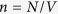 is the density of free electrons, and *μ* is their mobility.

Let a single electron is captured in the neutral deep center, and the total number of free electrons changes from *N* to *N* − 1. We shall try to evaluate the change of the resistance 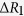 due to the change of the number of free electrons by 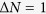, and also the total resistance change due to both effects: to electron capture and to screening of the negatively charge center due to electron capture. The electron capture and appearance of the Debye screened sphere are completely correlated events. If the sample is doped by shallow donors with density *n*_d_, which are completely ionized (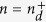), then the Debye screening length is^29^


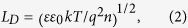


where 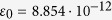 F/m is the permittivity constant, 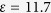 is the dielectric permittivity of silicon [20], *k* is the Boltzmann constant, *T* is the absolute temperature. Then the screened volume is


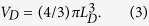


Comparison of characteristic parameters of the investigated silicon sample at *T* = 300 K is presented in the Table 1. It is seen that the volume of the Debye screened sphere at high density of charge carrier is many times smaller than the volume evaluated per one electron *V*/*N*, and it has a very small effect to the resistance fluctuations. But at charge carrier density 10^16^ cm^−3^ the volume of the Debye screened sphere about three times exceeds the volume *V*/*N*, and it has a large effect to the resistance fluctuations.

**Figure 2:**
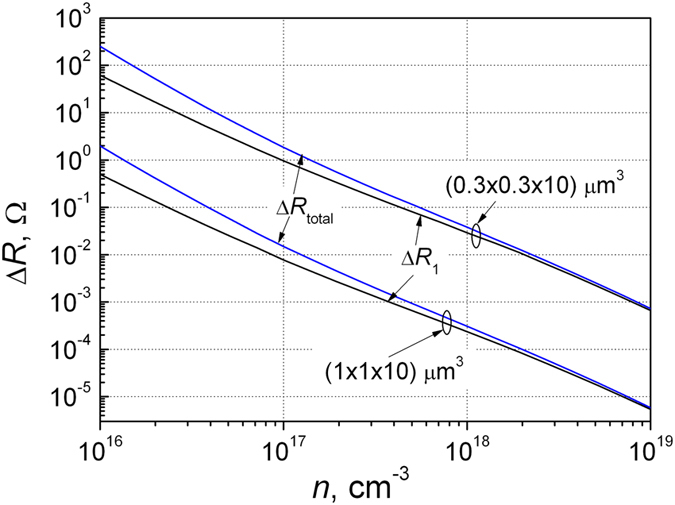
The resistance change due to single electron capture *ΔR*_1_ and the total resistance change including the charge screening effect *ΔR*_total_ dependences on the free electron density in the silicon sample for two volumes at room temperature.

Eliminating this screened (depleted) volume the total resistance fluctuation 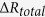 both due to the electron capture 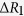 and due to the screening effect of this negatively charged center has been evaluated (Fig. 2). It is seen from this figure that resistance increase due to screening effect of captured electron is noticeable for a given volume when the electron density in the sample is smaller than 5·10^17^ cm^−3^, and at electron density 10^16^ cm^−3^ this increase is about 4 times larger than 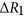 due to electron capture 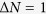. A larger intensity of RTS signal has been explained by assuming the capture of several electrons. Such capture center was called a giant trap. It was considered that this center acts as a gate to local conducting sample with *N* electrons. The gate operates by trapping and detrapping a single electron^21^. Thus, this effect can be explained on the base of the resistance change in small samples due screening effect of the captured electron.

The magnitude of the resistance change Δ*R* due to screening effect depends on the site where electron is captured: in the case when electron is captured near the surface defect, the change of Δ*R* due screening effect will be about two times smaller than in the inside of the volume.

### Evaluation of the resistance fluctuation spectrum

The power spectral density (PSD) of the resistance fluctuations due two parameters of random signal can be presented as^30^:


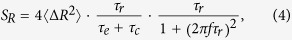


where the effective relaxation time


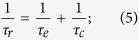


here *τ*_e_ is the average electron emission time, and *τ*_c_ is the average electron capture time in the defect level. The resistance fluctuations due to all capture centers *M*, can be presented by their superposition:


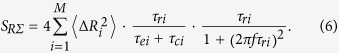


Dynamics of both electron emission times *τ*_e_ and electron capture times *τ*_c_ for different materials has been widely discussed in works^8^,^10^,^11^,^12^,^15^,^16^,^17^,^21^,^31^,^32^,^33^,^34^,^35^,^36^,^37^. As shown in^16^,^38^, the charge fluctuations in defects, even less than 100, with relaxation times *τ*_r_ arbitrarily distributed in a wide interval, up to large values, produce noise with 1/*f* type spectrum.

Now we shall try to estimate what minimum number M of defects (relaxators) with relaxation times distributed in wide time range needed for generation of noise with 1/*f* type spectrum (with uncertainty less than 5%), for example, in the frequency interval from 1 Hz to 1 MHz. For this purpose we shall analyze the expression:


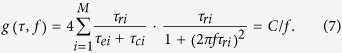


**Figure 3:**
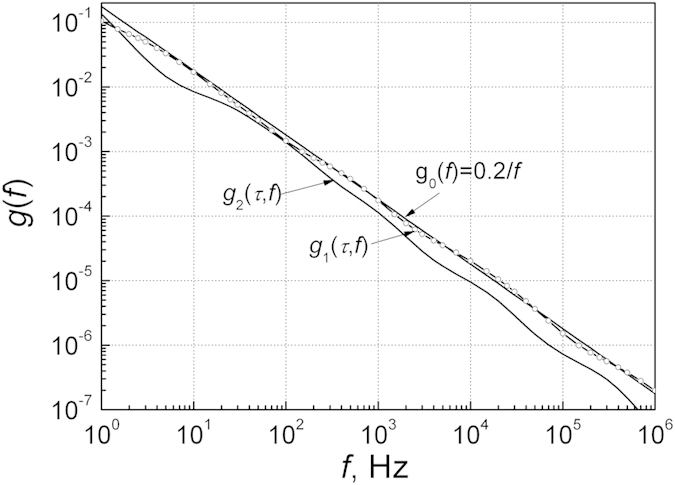
Modeled low frequency noise spectra with small number of widely distributed relaxation times *τ*_r*i*_. Function *g*_0_(*f*) = 0.2/*f* (linear line in logarithmic scale) shows the 1/*f* noise spectrum when the relaxation times are distributed as 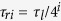, i. e., one-by-one of the relaxation time in every two octaves; *g*_1_(*τ, f*) (line with open dots) is the noise spectrum when relaxation times are arbitrarily distributed one-by-one in the range of every two octaves; *g*_2_(*τ, f*) (solid line) is the noise spectrum when relaxation times are arbitrarily distributed one-by-one in the range of every decade.

For simplicity we take that *τ*_ei_ = *τ*_ci_, when the Fermi energy coincides with the deep energy level of the defect. The simulated low frequency noise spectra are presented in Fig. 3. Function *g*_0_(*f*) = 0.2/*f* represents almost ideal 1/*f* law (uncertainty is less than 5%). It is obtained assuming that relaxation times *τ*_ri_ are distributed as 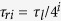, i. e., one-by-one relaxation time in every two octaves (here *τ*_*l*_ is the longest experimentally noticeable relaxation time in the investigated frequency range). It is needed only *M* = 15 relaxators providing the required relaxation times in order to generate 1/*f* noise in frequency range from 1 Hz to 1 MHz with high accuracy. In the case when these relaxation times are arbitrarily distributed one-by-one in every two octave range, the noise spectrum is presented by function *g*_1_(*τ, f*) (Fig. 3, line with open dots). It is seen that curve *g*_1_(*τ, f*) in average coincides with *g*_0_(*f*) = 0.2/*f*. The curve *g*_1_(*τ, f*) has only small waves or bumps comparing with *g*_0_(*f*). In the case when the relaxation times are arbitrarily distributed one-by-one in every decade range, the noise spectrum is presented by function *g*_2_(*τ, f*), which is lower and has a noticeable components of Lorentzian spectrum.

Thus, function *g*_0_(*f*) = 0.2/*f* can be used as a reference one for evaluating the Eq. 7. If in every two octave range there will be one-by-one independent relaxator with defined relaxation time, ones will obtain noise with 1/*f* spectrum with *C* = 0.2. In the case, when in every two octave range there will be in average number *K* of arbitrarily distributed independent relaxators, then ones obtain that *g*(*τ, f*) = 0.2 *K*/*f* with very small differences from 1/*f* law. So, the quantity *K* (not a total number of defects *M*) accounting the variations 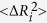 can be used for evaluation the number of relaxators causing the low frequency noise level in particular frequency range. Independent capture-emission events for every charge carrier produce the same resistance fluctuation Δ*R*_*i*_. There it must be pointed that function *g*(*τ, f*) only depends on the quantity *K* and the limits of the arbitrarily distributed relaxation times, but not depend on the physical mechanism causing these relaxation times, and it also does not depend on the volume of the sample. May be, it explains the fact that different materials and their devices generate low frequency noise with 1/*f*^γ^ type spectrum.

The resistance fluctuations cause the voltage fluctuations, when d.c. current flows through the sample. Usually for homogeneous samples 1/*f* noise is characterized by the Hooge parameter α as


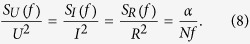


**Figure 4:**
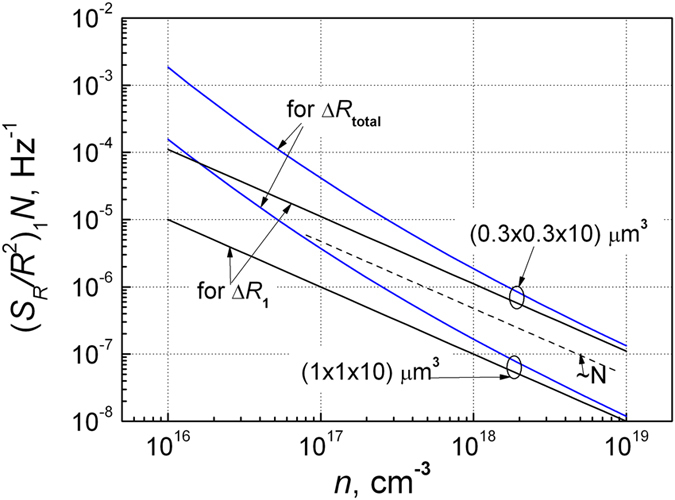
The normalized spectral density of the resistance fluctuations 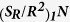 at *f* = 1 Hz due to single electron capture and due the total resistance fluctuations including the screening effect dependences on the electron density for two volumes of the samples.

In Fig. 4 it is shown the normalized resistance fluctuation spectral density 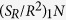 at *f* = 1 Hz (which in this case is proportional to parameter *α*) dependence on the free electron density in the sample. As it has been believed, this parameter due to electron capture process changes as Δ*R*_1_ ~ 1/*N* (or 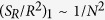), and for the total resistance fluctuation including the screening effect the Δ*R*_total_ dependence is steeper.

### Noise power spectral density relation with the charge carrier mobility

**Figure 5:**
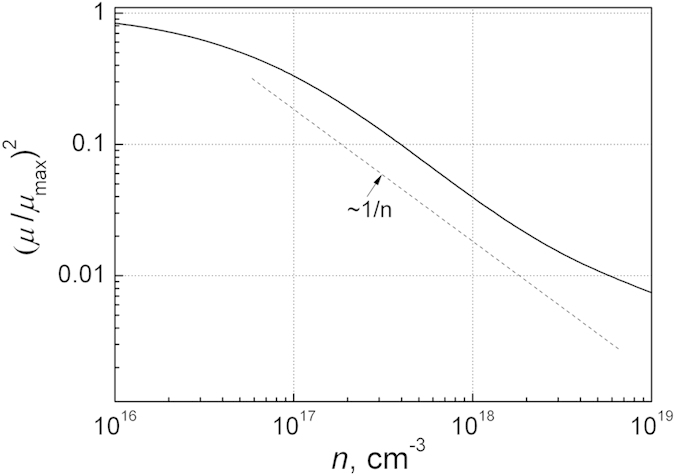
Relation between mobility and free electron density for silicon (according to data^27^,^39^).

For Ge, Si, GaAs and others materials in charge carrier density *n* range from 10^15^ cm^−3^ to 10^19^ cm^−3^ the relation between mobility and charge density can be approximated as^27^,^39^


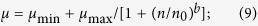


where *n*_0_ and *b* are the fitting parameters. For silicon this relation^27^,^39^ is presented in Fig. 5 in the form more convenient for further noise interpretation. It is seen that in the range of charge carrier density between 10^17^ cm^−3^ to 2·10^18^ cm^−3^ the square mobility changes in average as 1/*n*.

**Figure 6:**
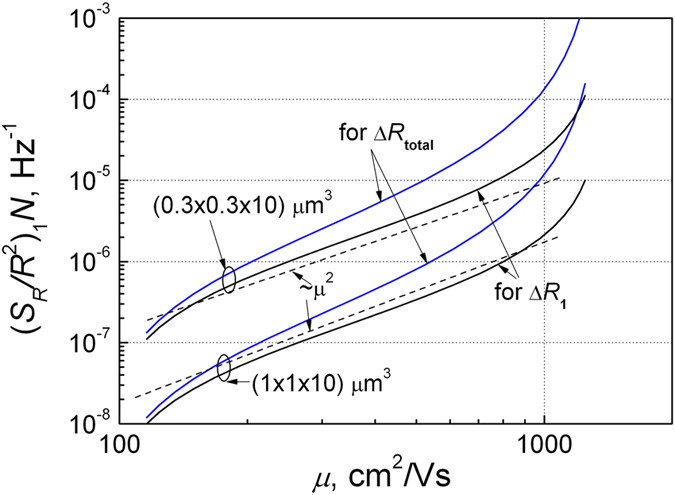
The normalized spectra density of the resistance fluctuations 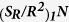 at *f* = 1 Hz due to single electron capture and due the total resistance fluctuations including the screening effect dependences on the electron mobility for two volumes of the sample.

Using the relation between the charge carrier density and their mobilities for silicon (Fig. 5), we represented the normalized spectra density of the resistance fluctuations 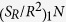 at *f* = 1 Hz dependence on the mobility. These results are shown in Fig. 6. The obtained data show that noise intensity in wide charge carrier density range is proportional to square mobility *μ*^2^. A steeper increase of the noise level at higher mobilities (*μ* > 1000 cm^2^/Vs) for silicon is due to the circumstance that at low charge carrier densities (*n* < 10^17^ cm^−3^) the mobility very weakly depends on the charge carrier density, but noise level is proportional to 1/*n*^2^ (Fig. 3). It is in agreement that parameter α values in this charge carrier density range are very scattered^4^,^14^,^25^. Thus, the proportionality of 1/*f* noise level to *μ*^2^ follows from the dependence of mobility on the free charge density (Fig. 5). When the normalized noise level 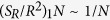 (Fig. 4), then it presentation versus *μ* in definite charge carrier range always gives approximately the proportionality to *μ*^2^. And conversely, if presented normalized noise spectral density is proportional to *μ*^2^, it means that 
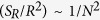
.

## Discussion

Low-frequency noise results for classical semiconductors such as Ge, Si, GaAs and others have been interpreted as charge carrier mobility fluctuations due to the lattice scattering^3^,^4^,^14^,^18^,^19^,^20^,^21^,^25^.


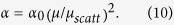


where *μ*_scatt_ is the mobility component caused by the lattice scattering. There were many unsuccessful attempts to explain the 1/*f*-type noise spectrum on the base of mobility fluctuations. As an argument there sometimes has been used that the measured noise level of scattered light provides an independent way of proving, and that the intensity of acoustic lattice modes varies with a 1/*f* spectrum^21^,^40^,^41^. Considering that acoustic waves (sound) velocity in silicon is about 7000 m/s^39^ (it depends on the direction), the phonon lifetime in the sample is very short. It seems that the nature of both resistance and acoustic phonon intensity fluctuations is the same. The charge carrier capture–emission process modulates the sample resistance, and as a consequence the charging–decharging of the neutral lattice center creates additional local electric field changes in the lattice and produces the phonon intensity fluctuations. Without it, if acoustic phonons would modulate the charge carrier mobility, then correlation length must be very large. But experiments show that correlation length for 1/*f* noise is smaller than 1 μm^42^.

So, the proportionality 1/*f* noise level to *μ*^2^ does not mean the mobility fluctuations: here mobility is only a presentation parameter (compare Figs 4 and 6), and that 1/*f* noise is defined by charge carrier capture–emission processes in the defect centers. It also does not mean that samples with the same number of the free charge carriers and capture centers, but with different mobilities of charge carriers, cause the larger 1/*f* noise level in the case of higher mobility of charge carrier.

Thus, the flicker noise spectrum in submicrometer samples with a few capture centers consists by several Lorentzian type spectra. Each such sample has various components of Lorentzian spectra caused by particular defects and on its position in the sample. Experimentally the decomposition of the 1/*f* spectrum into its constituent Lorentzian components has been demonstrated in^11^,^31^. The investigated devices produce random telegraph signals due to charge carrier capture in localized centers, and discrete resistance fluctuations are caused by individual carrier trapping events. In the larger samples having also larger number of defects, they obtain the noise with nearly 1/*f* spectrum^11^,^31^.

On the ground of Eqs. (6, 7, 8, 9, 10) and Figs 4, 5, 6 we can propose such general expression for 1/*f* noise caused by charge carrier capture-emission process:


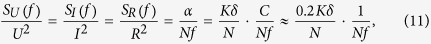


where


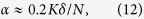


*K* is the average number of relaxators in the sample with arbitrarily distributed relaxation times in every double octave; *δ* is the factor accounting resistance fluctuation due to Debye screening effect (Fig. 4), for Si it can be approximated as 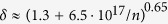. As seen from the Figs 4, 5, 6 α~1/*n*~(*μ*/*μ*_max_)^2^. Thus, the Eq. (11) reflects many experimental results of low frequency of noise for classical semiconductors. In the case, when some type of relaxators with particular relaxation times many times exceeds the average number of relaxators in the other relaxation time ranges, ones can observe the Lorentzian spectrum over 1/*f* noise. For example, the total number of defects in the sample responsible for 1/*f* noise in the measured frequency range from *f*_1_ = 1 Hz to *f*_2_ = 1 MHz is equal to *N*_t_ = *K*lg(*f*_2_/*f*_1_)/lg4 ≈ (6/0.6)*K* = 10 *K* (here the ratio (6/0.6) corresponds to the number of double octaves in the mentioned frequency range).

In conclusion, it can be pointed that our performed calculations data are in good agreement with experimental 1/*f* noise measurement results for semiconductors such as Ge, Si and GaAs^3^,^4^,^14^,^21^,^43^ both for small and large samples, and exclude the mobility fluctuations as the nature of 1/*f* noise. The proportionality 1/*f* noise intensity to mobility *μ*^2^ cannot be interpreted as the mobility fluctuations, because it is a circumstance of data presentation versus mobility due to particular relation between free charge carrier density and their mobilities. The presented calculation procedure let to one to evaluate the density of capture-emission defects in the sample from the low-frequency noise level. From this study also follows that with increasing the quality of the sample material the 1*/f* noise level decreases while the mobility may be almost constant.

## Methods

The calculation of the resistance fluctuations due to the charge carrier capture and emission process has been described as random telegraph signal, which power spectral density has been evaluated by using the Machlup method^30^. 1/*f* noise simulation has been done by summation of the Lorentzian type spectra with relaxation times arbitrarily distributed in the investigated frequency range. The effect of the negatively charged defect center due to capture of electron has been accounted as a screened depleted volume – the sphere with the Debye screening radius *L*_D_ (Eq. (2)). The value of the step Δ*L* for the evaluation of the resistance in the range 2*L*_D_ (Fig. 1) containing the Debye sphere has been chosen in such a way that the volume of the bar within the length 2*L*_D_ was equal to the sum of the volume of the Debye sphere and the rest volume part without this sphere.

## Additional Information

**How to cite this article**: Palenskis, V. and Maknys, K. Nature of low-frequency noise in homogeneous semiconductors. *Sci. Rep.* **5**, 18305; doi: 10.1038/srep18305 (2015).

## Figures and Tables

**Table 1:** Characteristic parameters of the investigated silicon sample (at *T* = 300 K).

Parameter	*n* = 10^16^ cm^−3^	*n* = 10^19^ cm^−3^
Total number of free electrons *N*	10^5^	10^8^
Sample volume (*V* = 1 × 1 × 10), μm^−3^	10	10
Volume per 1 electron *V*/*N*, μm^−3^	10^−4^	10^−7^
Debye screening length, μm	4.12·10^−2^	1.1·10^−3^
Debye sphere volume, μm^−3^	2.93·10^−4^	5.58·10^−9^
